# Ultradian rhythms in heart rate variability and distal body temperature anticipate onset of the luteinizing hormone surge

**DOI:** 10.1038/s41598-020-76236-6

**Published:** 2020-11-23

**Authors:** Azure D. Grant, Mark Newman, Lance J. Kriegsfeld

**Affiliations:** 1 The Helen Wills Neuroscience Institute, University of California, 175 Li Ka Shing Center, MC # 3370, Berkeley, CA 94720 USA; 2 Precision Analytical, McMinnville, OR 97128 USA; 3 Department of Psychology, University of California, Berkeley, CA 94720 USA; 4 Department of Integrative Biology, University of California, Berkeley, CA 94720 USA; 5 Graduate Group in Endocrinology, University of California, Berkeley, CA 94720 USA

**Keywords:** Computational biology and bioinformatics, Physiology, Systems biology, Biomarkers, Endocrinology

## Abstract

The menstrual cycle is characterized by predictable patterns of physiological change across timescales. Although patterns of reproductive hormones across the menstrual cycle, particularly ultradian rhythms, are well described, monitoring these measures repeatedly to predict the preovulatory luteinizing hormone (LH) surge is not practical. In the present study, we explored whether non-invasive measures coupled to the reproductive system: high frequency distal body temperature (DBT), sleeping heart rate (HR), sleeping heart rate variability (HRV), and sleep timing, could be used to anticipate the preovulatory LH surge in women. To test this possibility, we used signal processing to examine these measures in 45 premenopausal and 10 perimenopausal cycles alongside dates of supra-surge threshold LH and menstruation. Additionally, urinary estradiol and progesterone metabolites were measured daily surrounding the LH surge in 20 cycles. Wavelet analysis revealed a consistent pattern of DBT and HRV ultradian rhythm (2–5 h) power that uniquely enabled anticipation of the LH surge at least 2 days prior to its onset in 100% of individuals. Together, the present findings reveal fluctuations in distal body temperature and heart rate variability that consistently anticipate the LH surge, suggesting that automated ultradian rhythm monitoring may provide a novel and convenient method for non-invasive fertility assessment.

## Introduction

The fertility-awareness-method (FAM), a set of practices used to estimate the fertile and infertile days of the menstrual cycle, is challenging to implement and to study, and existing studies of its effectiveness are inconclusive^1^. However, an observation-based method of family planning or contraception has several potential benefits, including a lack of hormonal disruption, personalization, and relatively low cost. One challenge inherent to current FAM practices is the reliance on historical basal body temperature and symptom trends (e.g., breast tenderness, libido, cervical fluid) that can vary substantially by individual, within-individual from cycle-to-cycle^2^, and that provide predominantly retrospective information. The challenges of FAM have led the majority of those seeking to avoid pregnancy to adopt another form of contraception. Unfortunately, the most widely used method, female hormonal contraception, has short and long term risks for many users, including increased breast cancer rate^3,4^, luteal phase deficiency^5^, dysmenorrhea^5,6^, altered cognition^7,8^, and depressed mood^9,10^. These risks, combined with increasing recognition that many physiological systems vary in a structured manner across the menstrual cycle^11–14^, provide the impetus to develop FAM approaches that employ high-temporal-resolution, non-invasive measures of physiology.

The menstrual cycle is a continuous, rhythmic succession of endocrine, ovarian, and uterine events. Briefly, the cycle begins with onset of menstruation, followed by rising levels of estradiol, follicular maturation, and proliferation of the uterine lining^15,16^. Ovulation, which is triggered by numerous factors including estradiol, a surge of luteinizing hormone (LH), the presence of a mature Graafian follicle, and likely time of day^17^, frequently occurs between 1/2 and 3/4 of the way through the cycle in humans^18^. Other physiological systems, including metabolism^19, 20^ and autonomic balance^21^, fluctuate with the menstrual cycle. An individual is mostly likely to become pregnant during the time leading up to, and shortly past, the ovulation event, making identification of this peri-ovulatory period central for the successful use of the FAM. Although high-frequency hormone measurements (e.g., daily estradiol from blood or urine) and ultrasound can provide information on when an LH surge and subsequent ovulation are likely to occur, such measurements are both laborious and expensive, limiting their widespread utility. Furthermore, at home tests available for measuring supra-threshold LH concentrations provide retrospective rather than prospective information about this event. Ideally, new methods of fertility awareness would accurately indicate the approaching peri-ovulatory period via relatively inexpensive and non-invasive means^22^. This study aimed to develop such a preliminary indicator for future, larger scale investigation.

The premise of the present investigation is that the presence of structured changes to peripheral biological rhythms across the menstrual cycle may allow for anticipation of the LH surge. Such a finding would further support the notion that the state of one system (e.g., reproductive) can be inferred via measurements of another (e.g., autonomic or metabolic)^14,23,56^. Perhaps the most consistent biological rhythmic changes across the menstrual cycle occur at the few hour (ultradian rhythm, UR) timescale^14,23–26^. Most elements of the hypothalamic-pituitary-ovarian axis, including gonadotropin releasing hormone (GnRH)^27–29^, LH ^30–32^, FSH^33–36^, estradiol^30,37^, progesterone^30,31,38–41^, and testosterone^42^ show URs that are coordinated with menstrual phase^14^. Across species, timeseries of these neuropeptides and hormones exhibit an increase in ultradian frequency and inter-hormone coupling strength leading up to ovulation^29,31^ and a decrease in ultradian frequency and stability in the luteal phase^29–32,37,40,41,43^. Additionally, peripheral measures of distal body temperature (DBT) and heart rate variability (HRV) reflect the activity of reproductive^44–46^, autonomic^21,23, 47–52^, and metabolic systems^23,53–55^ and show both URs and menstrual rhythms^44^. These peripheral and endocrine measures are proposed to operate as coupled oscillators at the ultradian timescale. Assessment of these peripheral measures could, therefore, potentially enable endocrine status assessment via timeseries analysis^14,23,56^.

Recent animal work supports the idea that non-reproductive measures can be used to anticipate reproductive status. In rodents, the wavelet power of core body temperature URs exhibits a trough on the day of ovulation^12,13^. The translational capability of this method is supported by the association of gross timescale changes in DBT, heart rate (HR), and HRV by menstrual phase^11,19,44,45,53,57–61^. However, it is unknown if human ovulatory cycle phase is associated with patterns of rhythmic change in non-reproductive outputs. Although the specific factors responsible for the changes in frequency of reproductive URs across non-human mammalian ovulatory cycles are not well understood, their consistency of change across species of widely varying cycle lengths suggests a concerted role in ovulatory cycle function^14^. Finally, although the structure of some circadian rhythms (~ 24 h; CRs) is altered in the luteal phase, with estradiol acrophase advancing, and REM sleep exhibiting a modest decrease; structured sleep and circadian changes are not generally observed during the peri-ovulatory period^62^. As both URs and CRs are tightly regulated across systems, monitoring their structure may enable more accurate assessment of reproductive state than is possible using infrequently collected data (e.g., 1 temperature time point per day)^26,63,64^. Wearable devices offer unprecedented ease of collecting the continuous, longitudinal data needed to assess URs and CRs across the menstrual cycle^65–68^. To determine if rhythmic structure exhibits reliable changes leading up to the LH surge, we used a wearable device (the Oura Ring) to monitor DBT, sleeping HR, sleeping HRV (root mean square of successive differences; RMSSD), sleep timing, and duration. If endocrine, metabolic, and autonomic rhythms are sufficiently coupled at the ultradian and circadian timescales, then coordinated patterns should be observed across measures and potentially across the menstrual cycle. Such patterns would contribute to a growing body of work in “network physiology”^69,70^, which proposes that changes among endocrine, metabolic, and autonomic outputs are coupled under real world conditions. As mentioned above, implicit in this hypothesis is that one could infer the state of one system via measurements of another. Anticipation of female reproductive events is a test of the network physiology framework with potential for rapid translation.

## Results

### Demographics

Findings are reported for individuals with premenopausal cycles (*n* = 20, *n* = 45 cycles, 2–3 cycles per individual) or perimenopausal cycles as (*n* = 5, *n* = 10 cycles, 2 cycles per individual) as defined in the Methods. Individuals who became pregnant (*n* = 3) during the study were excluded from the analyses. All premenopausal participants experienced menses, 1 or more supra-threshold LH readings per cycle, and a subsequent, sustained rise in temperature deviation during all cycles. See Table 1 for participant age, ethnicity, cycle length, LH surge length, LH surge onset timing, LH surge onset relative to estradiol (E2) peak and progesterone rise, and percent of individuals with regular cycles. Some variability was observed in the day of LH surge onset relative to day of E2 peak(s), as previously reported^71^.

**Table. 1:** Demographics of the QCycle cohort, including *n* values, age, ethnicity, and hormonal cycle characteristics.

Factor	Premenopausal	Perimenopausal
Number of participants	20	6
Number of cycles	45	10
Age range, mean (STDEV) *years*	21–38, 32 (4)	48–60, 55 (5)
Ethnicity (%)	Caucasian: 94; African American 6	Caucasian: 100%
Cycle length	25–36, 27.78 (4.16)	22–50, 28.7 (8.87)
LH surge length range, mean (STDEV) *days*	1–5,1.95 (1.2)	N/A; LH tonically high
LH surge onset day	10–29, 15.75 (3.4)	N/A; LH tonically high
LH surge onset relative to E2 peak in *days*	0–4, 1.67, (1.38)	N/A ; LH tonically high
LH surge day relative to progesterone rise *day*(*n* = 21)	0–5, 1.14, (1.95)	N/A; LH tonically high
Regular cyclers (%)	88	0

### Premenopausal and perimenopausal estradiol, luteinizing hormone and progesterone metabolites

**Figure 1:**
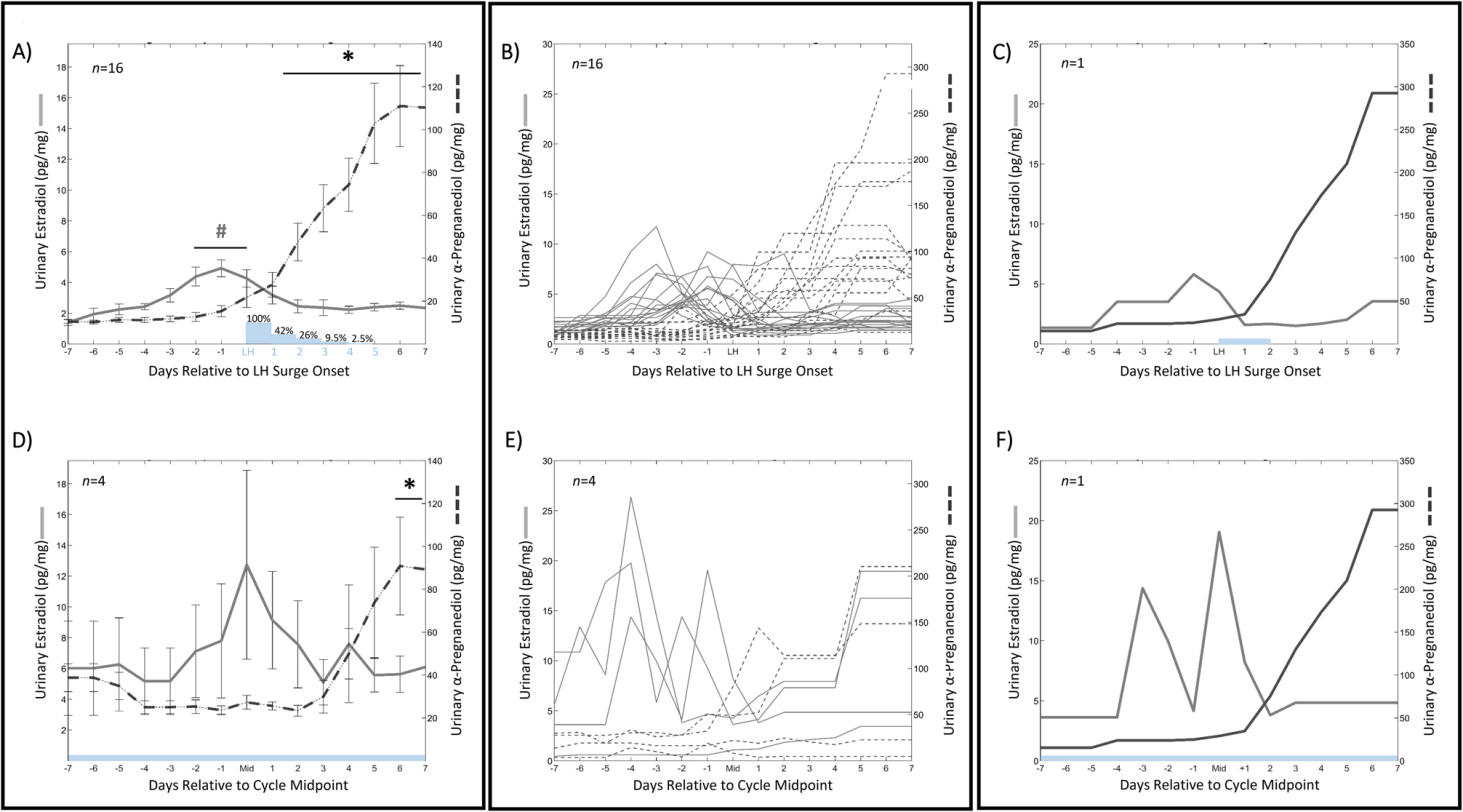
Ovulatory & Perimenopausal E2 and αPg. Linear plots of premenopausal (**A**–**C**) and perimenopausal (**D**–**F**) E2 and αPg. Mean ± standard deviation E2 (solid) and αPg (dashed) concentrations for premenopausal (**A**) and perimenopausal cycles (**D**) within one week of LH surge onset (N = 16 out of 45 premenopausal cycles, and N = 4 out of 10 perimenopausal cycles). # Indicates significantly elevated pre-LH E2 concentrations (premenopausal *p* = 5.5 × 10^–5^; perimenopausal non-significant *p* = 0.391), and * indicates significantly elevated αPg after LH surge onset (premenopausal *p* = 4.71 × 10^–31^; perimenopausal, *p* = 0.028). Blue bars and text and indicate percent of cycles showing an LH surge a given number of days after onset, beginning on the day marked “LH” (e.g., 26% indicates that 26% of individuals were still surging on the 3rd day after LH surge onset). Representative E2 (gray) and αPg (black) from premenopausal (**C**) and perimenopausal (**F**) individuals relative to LH surge onset, and cycle mid-point, respectively.

Participants monitored LH for all 55 cycles, whereas daily urine samples were collected by 20 women (*n* = 16 premenopausal, *n* = 4 perimenopausal) for the measurement of E2, α-Pregnanediol (αPg) and β-Pregnanediol (βPg). E2, *α*Pg and βPg were collected to confirm that hormone concentrations were within healthy ranges for pre-menopausal women and that LH surges were followed by a rise in progesterone metabolites. For all 16 cycles, estradiol, αPg and βPg fell within normal ranges, with a pre-LH rise in E2 (2 days prior to LH onset through LH onset day were significantly greater than all other days, *p* < 0.01 in all cases). Likewise, LH surge onset was concomitant with a significant rise in αPg (*p* < 0.05 on LH onset, and < 0.01 6 days after LH onset and thereafter) (Fig. 1A−C) and βPg (*p* < 0.005 on LH onset, and < 0.001 3 days after LH onset; data not shown for βPg). These hormonal changes were associated with a rise in temperature deviation above zero and a non-significant elevation of breathing rate around LH surge onset (Supplemental Fig. 1E–F). Consistent with previous findings^71^, LH surge length was variable, with 42% of individuals exhibiting supra-threshold LH concentrations 2 days following surge onset, falling to 26% of individuals 3 days after surge onset (Fig. 1A). LH was tonically supra-threshold in perimenopausal women (*n* = 10 cycles, Fig. 1D). Perimenopausal individuals exhibited a significant increase in αPg and βPg only 6 days after midcycle (*p* < 0.05; data not shown for βPg), and a trend toward elevation of E2 prior to mid cycle (*p* = 0.176) (Fig. 1D−F).

### Ultradian power of DBT, HRV, and LH surge onset

**Figure 2:**
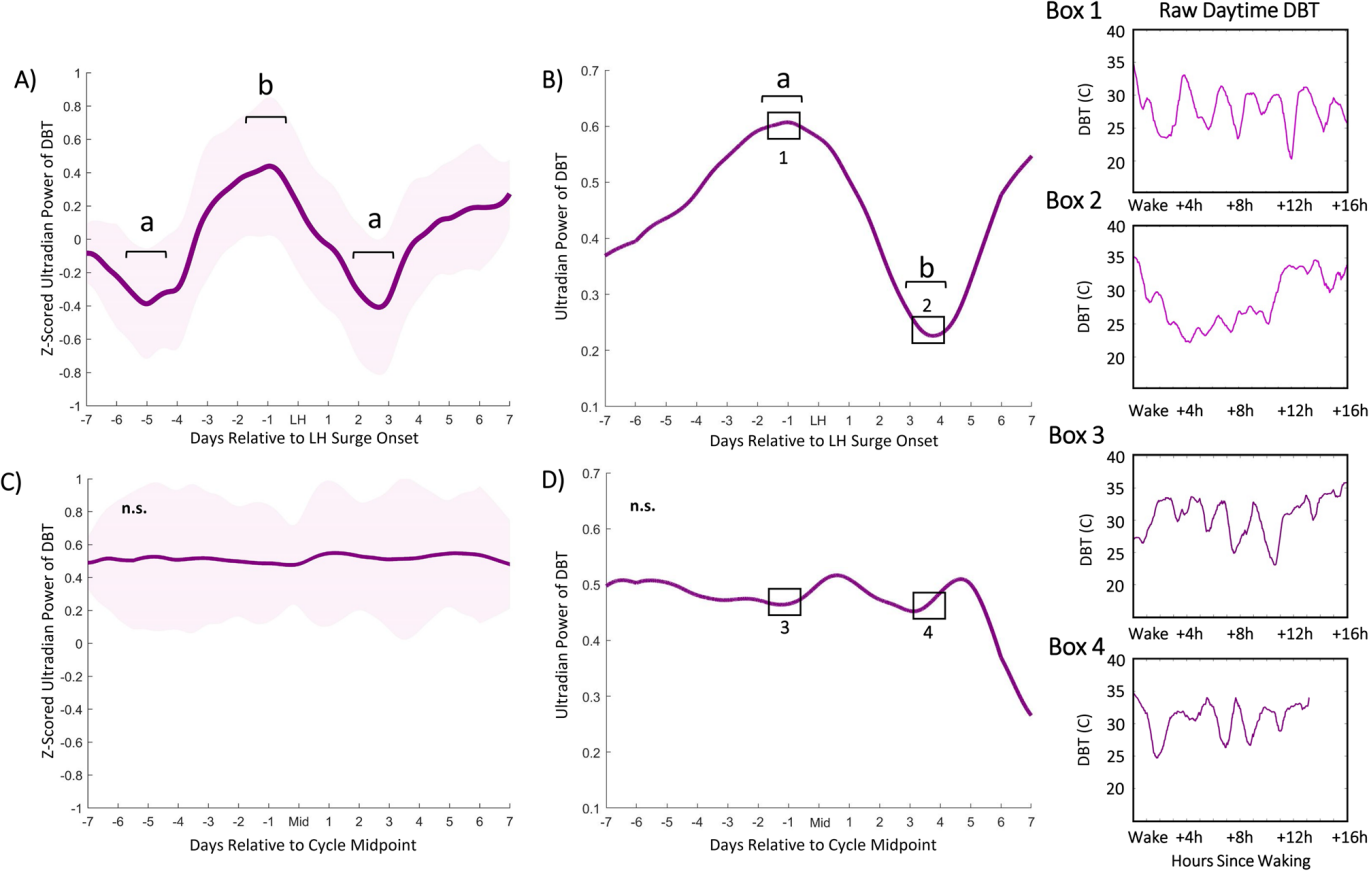
Ultradian power of DBT anticipates LH surge onset. Mean DBT ultradian power (z-scored) ± standard deviation for premenopausal cycles (**A**) within one week of LH surge onset and perimenopausal cycles (**C**) within one week of mid cycle. DBT UR power peaks exhibit an inflection point 5.82 (± 1.82) days prior to LH onset, a peak a mean of 2.58 (± 1.89) before LH onset on average and a subsequent trough a mean of 2.6 (± 1.02) days after surge onset (χ^2^ = 5.66, *p* = 0.0174). Perimenopausal UR power shows no conserved peaks and troughs (χ^2^ = 0.37, *p* = 0.5354, for same comparisons). Representative individual example of raw DBT ultradian power within one week of LH surge onset in premenopausal (**B**) and within one week of mid cycle in perimenopausal (**D**) cycles. Black squares in (**B**) and (**D**) correspond to Boxes 1 & 2 and Boxes 3 & 4, respectively. Boxes show linear waking DBT from which ultradian power in B and D were generated; these days were selected to visually illustrate days of relatively high and low ultradian power in premenopausal cycles, and the same two days in perimenopausal cycles.

**Figure 3:**
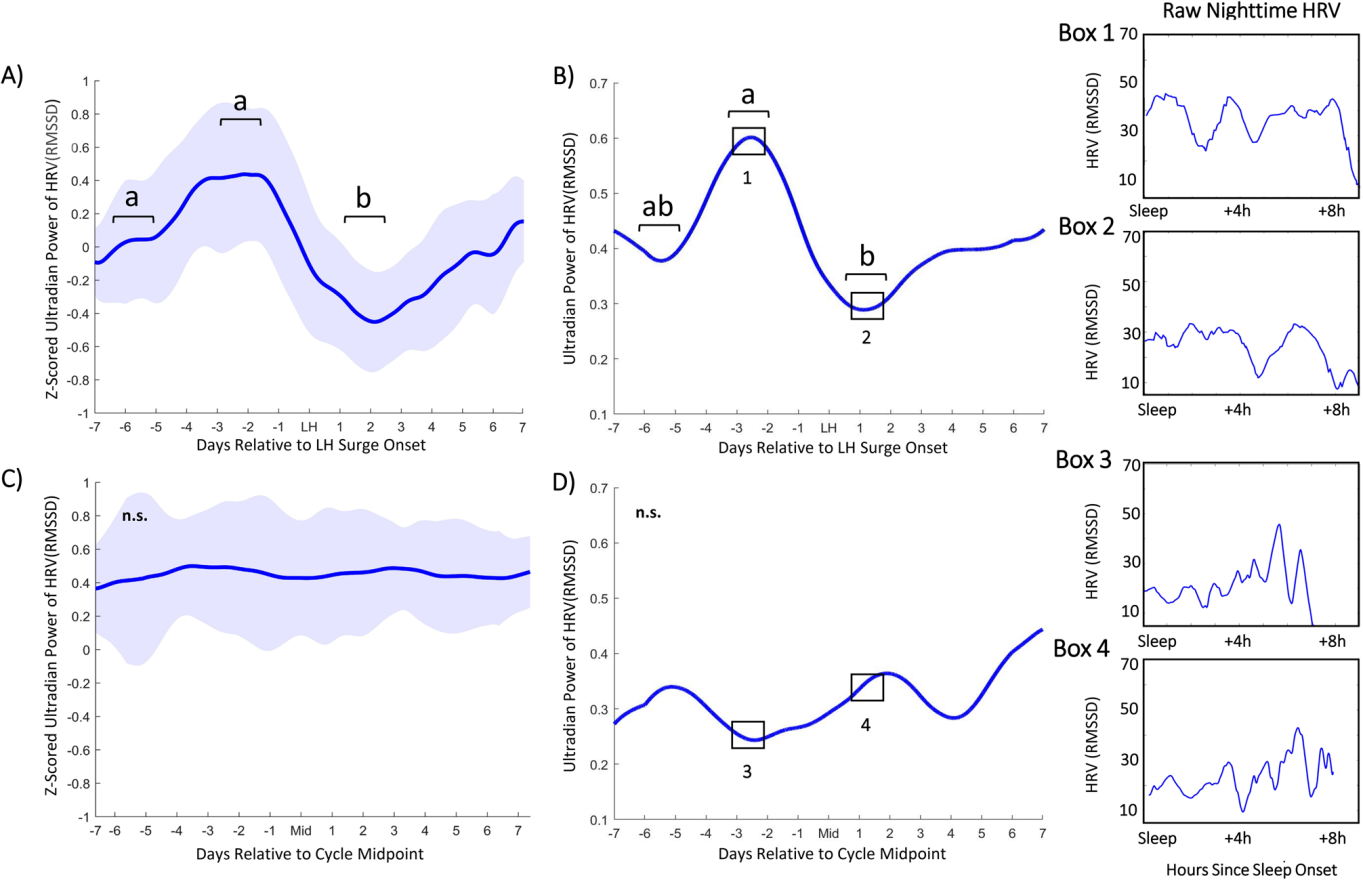
Ultradian power of HRV (RMSSD) anticipates LH surge onset. Mean HRV (RMSSD) ultradian power (z-scored) ± standard deviation for premenopausal cycles (**A**) within one week of LH surge onset and perimenopausal cycles (**C**) within one week of mid cycle. Ultradian HRV (RMSSD) power inflects an average of 5.82 (± 1.53) nights prior to LH surge onset, exhibits a subsequent peak an average of 2.58 (± 1.59) days prior to the surge onset and a trough an average of 2.11 (± 1.27) days after surge onset. (χ^2^ = 4.91, *p* = 0.034,). These stereotyped changes are not present in perimenopausal cycles (χ^2^ = 0.4797, *p* = 0.57). Representative individual example of HRV (RMSSD) ultradian power within one week of LH surge onset in premenopausal (**B**) and perimenopausal (**D**) cycles. Black boxes in (**B**) and (**D**) correspond to Boxes 1 & 2 and Boxes 3 & 4, respectively. Boxes show linear sleeping HRV (RMSSD) signal from which (**B**) and (**D**) were generated, illustrating days of relatively high and low ultradian power in ovulatory cycles, and the same two days in perimenopausal cycles.

Ultradian (2−5 h) power of daytime DBT exhibited a stereotyped pattern preceding LH surge onset in premenopausal (Fig. 2A,B), but not perimenopausal (Fig. 2C,D), cycles. Ultradian DBT power exhibited an inflection point a mean of 5.82 (± 1.82) days prior to LH surge onset and a subsequent peak a mean of 2.58 (± 1.89) days prior to the surge onset. A second trough in UR power occurred a mean of 2.06 (± 1.02) days after surge onset (χ^2^ = 5.66, p = 0.0174,). These stereotyped changes were not present in perimenopausal cycles (χ^2^ = 0.37, p = 0.5354, for the same comparisons) (Fig. 2C). Ultradian power of sleeping HRV (RMSSD) also exhibited a stereotyped fluctuation preceding LH surge onset in premenopausal (Fig. 3A,B), but not perimenopausal (Fig. 3C,D), cycles. Ultradian HRV (RMSSD) power showed an inflection point with a mean of 5.82 (± 1.53) nights prior to LH surge onset, a subsequent peak with a mean of 2.58 (± 1.59) nights prior to the surge onset and a trough a mean of 2.11 (± 1.27) days after surge onset. (χ^2^ = 4.91, p = 0.034). These stereotyped changes were not present in perimenopausal cycles (χ^2^ = 0.4797, p = 0.57) (Fig. 3C). Ultradian power of HR and circadian power of DBT did not show a significant pattern of change preceding the LH surge (χ^2^ = 0.3 and 1.12, p = 0.581 and 0.2899), nor mid cycle in perimenopausal individuals (χ^2^ = 0.02 and 1.65, p = 0.8798 and 0.1984, respectively) (See Supplemental Figs. 2 and 3). No significant trends were observed in sleep metrics captured once per night (See Supplemental Fig. 1). Linear means of nightly HR and HRV, and continuous DBT did not yield consistent patterns of change relative to surge onset or perimenopausal midcycle (Supplemental Figs. 4–6).

**Figure 4:**
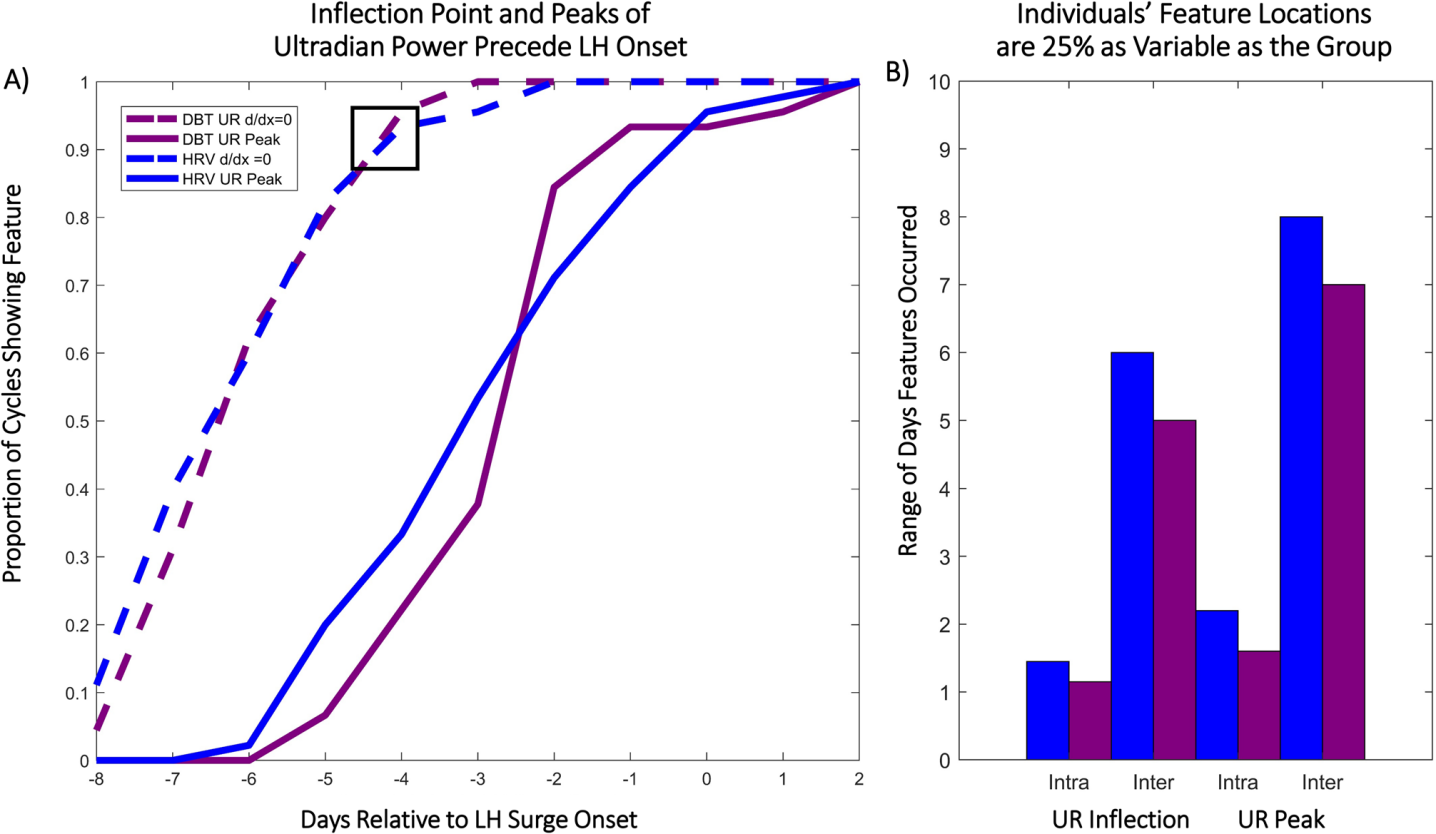
Inflection Points and Peaks of Ultradian Power Anticipate the LH Surge Within and Across Individuals. (**A**) Cumulative histogram indicates the proportion of cycles showing an inflection point on a given day relative to LH surge onset (blue = HRV (RMSSD), maroon = DBT, dashed = inflection point, solid = subsequent peak). Box indicates that on day LH—4, ~ 90% of individuals had shown the HRV and DBT first inflection point. (**B**) Intra-vs. inter-individual range of days over which inflection points (“UR Inflection”) and subsequent peaks (“UR Peak”) of ultradian DBT and HRV power occurred. The range intra-individual range (2–3 cycles per individual) is 25% the size of the inter-individual range (45 total cycles).

### Inflection point and subsequent peak of DBT and HRV ultradian power anticipate LH surge onset

In premenopausal women, the first inflection point of DBT and HRV (RMSSD) UR power occurred between -8 and -2 days prior to surge onset, whereas the subsequent peak in UR power for both metrics occurred between -6 days before to 2 days after LH surge onset (Fig. 4). 85% of cycles exhibited the first inflection point by 4 days prior to the surge, with 100% showing this inflection by 2 days prior to the surge. The peak of UR power occurred at least 1 day prior to the surge in 82% of cycles. Together, these inflection points and subsequent peaks in UR power of HRV (RMSSD) and DBT uniquely anticipated the LH surge days before its onset (see "Discussion" for potential relevance to fertile window).

## Discussion

The present findings reveal stereotyped fluctuations in DBT and HRV (RMSSD) UR power that anticipate 100% of LH surge onsets, a key component of female health and fertility. By contrast, changes in DBT circadian rhythm power were not predictive of the LH surge, suggesting that URs are uniquely coupled to the pre-ovulatory time of the menstrual cycle. Likewise, discrete, nightly behavioral and physiological measures did not anticipate the surge, suggesting that continuous measures of physiological output provide signals more amenable to LH surge anticipation. Finally, these features did not occur stereotypically in perimenopausal cycles with respect to mid cycle. These findings point to peripheral URs as oscillations that are coupled to menstrual cycle physiology and that have the potential to contribute to the development of tools for estimating the female fertile window.

Although the underlying physiological mechanisms that lead to systematic changes in UR power in DBT require further investigation, much is known about general changes in body temperature across the menstrual cycle. Estrogens lower, and progestins raise, body temperature^53,72^. Accordingly, body temperature reaches a minimum, with minimum core circadian amplitude, during the late follicular phase and rises in the core, mouth, and skin following ovulation^73^. Body temperature also broadly reflects metabolic rate, which is elevated in the late follicular and luteal phases^74^. In mice, the structure of core temperature URs allows for the detection of female reproductive state, with a high plateau of temperature and trough of UR power during the active phase indicative of the LH surge and ovulation^13,75^. Most human studies to date have focused on core temperature, measured via an ingestible device that travels through the GI tract^73^, intravaginal or rectal sensor^76^, or oral thermometer^60^. However, ultradian, circadian, and ovulatory rhythms in temperature are readily observed at the periphery, providing several advantages: (1) DBT has higher amplitude fluctuations than core body temperature, making URs and CRs easier to detect^77^, (2) changes in DBT may correlate with sleep stage^78^, and (3) DBT is in circadian antiphase to core temperature, but shows the same general trend across the menstrual cycle, suggesting comparable reliability^77^. It is possible that rising UR power of DBT before the LH surge reflects higher UR power of reproductive hormones during this time.

Whereas body temperature is the most commonly used non-hormonal output in menstrual cycle tracking, previous studies have found that HRV also changes by cycle phase and may therefore be a candidate for surge anticipation^79^. Parasympathetic input to the heart dominates during the follicular phase, lowering resting heart rate and elevating HRV (RMSSD)^57^. Sympathetic input to the heart dominates during the luteal phase, elevating heart rate and depressing HRV (RMSSD)^11,57^. Consequently, HRV (RMSSD) varies ~ 10 ms from the follicular to the luteal phase^11^, with a marked decrease in the latter portion of the cycle^52^. These fluctuations may be more difficult to detect during a short daytime recording window, and are impacted by daytime activities, making sleep an ideal window over which to look for unmasked features^45^. Natural negative controls illustrating reproductive and metabolic influences on HRV patterns are that (1) LH pulsatility is disrupted in obese and diabetic women^80,81^, and (2) mid cycle and luteal fluctuations in HRV are absent in polycystic ovarian syndrome (PCOS), a leading cause of female infertility^58,59^. In the present study, sleeping HRV (RMSSD) UR power rose in the late follicular phase, peaked near the LH surge, and dropped sharply before rising into the early luteal phase. Although the present study lacks sufficient power to evaluate other potential patterns that may be relevant to the menopausal transition, the preliminary absence of comparable features in perimenopausal individuals suggests that this group deserves further study. Together, signal processing of DBT and HRV could yield actionable information for individuals and clinicians wishing to estimate the “fertile window”. However, there are several challenges inherent to accurately defining the female fertile window.

The fertile window (the time during which a woman may become pregnant) depends upon many factors, including (1) the timing of the LH surge, (2) the subsequent time of the release of the ovum or ovulation, (3) the presence of a viable corpus luteum releasing adequate progesterone^38^, (4) the duration of time sperm can survive in the female body, which is dependent both on sufficient number and quality of sperm and on the appropriate vaginal environment (e.g., pH)^22,82^, and 5) quality of the uterine environment. Most investigations report the highest probability of fertility as the 5 days preceding ultrasound-determined day of ovulation (USDO)^83^, but actual days on which an individual may become pregnant are much more variable, with pregnancy occurring up to 11 days prior to ovulation to 5 days after ovulation^71^.

Some of the reported variability in the fertile window likely results from discrepancies in language used to describe both human ovulation and the fertile window itself^84^. Despite their namesake, home “ovulation tests” that identify supra-threshold LH concentrations do not measure ovulation, which may occur many days after and occasionally a few days *before* LH surge onset^71^. Despite this variability, the fertile window is often treated as predictable, with definitions including the 5–6 days prior to the LH surge as a proxy for ovulation^85^, the first day of slippery clear cervical fluid through LH surge onset^86^, the total days of slippery clear cervical fluid^87^, day 10–17 of the cycle^88^, and retrospective measures of salivary ferning^85^, basal body temperature^64,89^, and progesterone metabolites (e.g.,^90^). Today, many online and app-based ovulation prediction algorithms are validated using day of cycle or LH data alone, in the absence of hormone measures or USDO^2,83,84,91^. Additionally, extant data sets regularly report excluding 20–50% of collected data due to cycle irregularities, without determining if given cycles were hormonally aberrant^71,92–94^. Together, the confounding of the LH surge with ovulation and the variable criteria used to define the fertile window make it difficult to accurately determine the variance, and contributors to variability, of fertility relative to the LH surge or ovulation. Despite these discrepancies, the possibility that UR features anticipate the onset of the LH surge by a few to several days suggests applicability for family planning. When one considers the additional time between LH surge onset and ovulation, these features may anticipate much to all of the fertile window. If confirmed in larger cohorts, this method would constitute the earliest method of predicting a definitive event at any point within the fertile window.

Open source, non-invasive methods for predicting the LH surge as a marker of likely future ovulation are not currently available^71^, but the present findings indicate that the onset of the LH surge may be anticipated days in advance by automated detection of changes in ultradian power of DBT and HRV (RMSSD). These changes consistently anticipate LH surge onset in women of a variety of ages, cycle lengths, surge timing and duration. The frequency band of 2–5 h examined in the present investigation was not specifically selected for the present group of participants but chosen based on the peak frequency band observed across physiological systems^14,25,56^, suggesting potentially broad applicability. Due to the high demand for accurate methods of fertility assessment, such novel methods carry the responsibility to clearly report the aspects of reproductive physiology that are detected and the methods by which detection is achieved once algorithms are tested on large populations^68,84,91^.

Future work will determine the extent to which these ultradian rhythm-based methods of menstrual cycle monitoring generate accurate predictions in larger, more diverse cohorts. In particular, the study of a greater number of cycles within individuals may enable personalization of relevant features. With these data, methods such as empirical mode decomposition for selection of tailored ultradian bands, or machine learning based methods for assessment across many different features at once, may result in greater specificity or longer predictive windows. These features could potentially be used on their own, with minimal user input (e.g., tracking of dates of menstruation), or in combination with other FAM methods. Ideally, such methods could be widely employed on wearable devices such as the Oura Ring, or on future generations of convenient and precise body temperature and HRV sensors. Together, these findings may guide further research aimed at understanding how hormones, metabolism, and the autonomic nervous system temporally interact; and may aid the development of open-source, non-invasive methods of fertility awareness.

## Methods

### Ethical approval

This study and all procedures were approved by the Office for the Protection of Human Subjects at the University of California, Berkeley. All participants gave informed consent. All research was performed in accordance with relevant guidelines and regulations.

### Participants and recruitment

Participants were recruited from the Quantified Self community, a global group of individuals interested in learning through self-measurement^95,96^. Individuals attended a prospective discussion about the project at the 2018 Quantified Self meeting in Portland, Oregon and contacted the experimenter via email if interested in participating. Prospective participants were contacted to discuss study structure, risks and benefits, and to review the informed consent form. Once informed consent was obtained, participants were instructed to complete an introductory questionnaire with their age, cycling status (regular, irregular, recovering from hormone/IUD use, perimenopausal, menopausal), and historical LH surge day(s), if known. Contact information was collected for the purposes of communication and delivery of study materials. Data from pregnancies (*n* = 3) that overlapped with the study were excluded from these analyses. Participants had not taken hormonal contraception within the prior year and did not have any known reproductive medical concerns. There were no age or parity restrictions, consistent with the principles of participatory research^96,97^. See Table 1 for participant demographics.

### Study design

Each of the 28 (*n* = 20 premenopausal, *n* = 5 perimenopausal, *n* = 3 premenopausal and became pregnant) participants collected 2 to 3 cycles of data for analysis. For all cycles, the Oura Ring, a DBT, HRV (RMSSD), HR, and sleep sensor, was worn continuously on the finger, as previously described^65,98^. For all cycles, LH was monitored via urinary test strips (Wondfo Biotech Co., Guangzhou, China) from day 10 (with first day of menstruation considered day 1) until a positive reading was detected, and subsequently until 2 days after LH fell below the limit of detection (see below for details on the *Urinary Hormone Assay, Luteinizing Hormone*). Of the 55 total cycles collected (45 = premenopausal, 10 = perimenopausal), 20 were paired with daily, morning urine tests for E2, αPg and βPg, the major urinary progesterone metabolites (Precision Analytical, McMinnville, OR). This study was designed using the principles of participatory research^96,99^ in which individual participants maintain control of their own data prior to anonymization and came to the project with personal questions that could be answered with the data to be collected. All participants received a copy of their Oura Ring data.

### Data collection and management

HR, HRV (RMSSD), DBT, sleep onset, sleep offset, sleep duration, breathing rate, and nightly temperature deviation (described briefly below) were collected using the Oura Ring (Oura Inc., San Francisco, CA; Oura Health Oy, Ltd., Oulu, Finland). The Oura Ring is a small, wireless sensor worn on the finger. By using an LED light source and LED sensor to measure reflection off the skin above the radial artery of the finger, the Oura Ring calculates HR, HRV (RMSSD), and breathing rate. The ring also contains 3 thermistors for detection of DBT. DBT is measured 24 h a day (binned in 1-min intervals). To avoid artifacts associated with activity, HR, and HRV (RMSSD) are only measured during sleep (binned in 5-min intervals), limiting our analyses of HR and HRV (RMSSD) to the sleeping period. All other metrics are calculated once per night. Briefly, the body temperature deviation for each night is the moving mean of nightly temperature between 10:00 pm and 8:00 am, minus the mean temperature of the previous 20 days. Oura Rings were loaned to the group by Oura Inc.

The Oura Ring can be connected to a mobile phone application, Oura, via Bluetooth. At the start of the study, each participant downloaded the Oura application from either the Google Play Store (Google Inc., Mountain View, CA) or the Apple App Store (Apple Inc, Cupertino, CA) to their mobile phones and created an Oura account. Participants were able to view their own data provided by the application throughout the study. Participants were asked to synchronize data from the ring to the application each morning. Uploaded data was automatically transferred via the internet to the study database in the Oura cloud service. In order to access data from the cloud, data were imported into the Open Humans^100^ framework, which provides encrypted, password protected data access to researchers, with the participants’ revocable consent. In addition to data collected by the Oura Ring, participants uploaded personal spreadsheets that tracked days of menstruation, days of LH tests and results, days of urine collection, and notes (e.g., forgot to wear the Oura Ring) to Open Humans. Participants could opt out of the study and remove their data at any time. Data were anonymized by the researchers for analysis. Data, once anonymized at the end of the study, remained in the data set.

### Hormone assays

For the assessment of E2, αPg and βPg, participants collected daily, first-morning urine samples across a cycle according to manufacturer’s instructions (Precision Analytical, Willamette, OR). Briefly, a standardized piece of filter paper with an attached label was submerged in the urine sample and dried for 24 h. Filter paper was then frozen at ~ -18 C in participants’ home freezers until analysis. E2, αPg, and βPg were analyzed using proprietary in-house assays referred to as Dried Urine Testing for Comprehensive Hormones (DUTCH) on the Agilent 7890/7000B GC–MS/MS (Agilent Technologies, Santa Clara, CA, USA). The equivalent of approximately 600 μl of urine was extracted from the filter paper using acetate buffer. In the first week of the cycle, and from 3 days after LH surge completion until the end of the luteal phase, samples were pooled every 2 days (a third day was pooled at the end of cycles in instances where the total number of remaining days after the surge was odd). Urine samples were extracted and analyzed as previously described, with previously established ranges of hormone concentrations expected in urine by phase of cycle and during menopause^90,101^. Briefly, creatinine was measured in duplicate using a conventional colorimetric (Jaffe) assay. Conjugated hormones were extracted (C18 solid phase extraction), hydrolyzed by Helix pomatia and derivatized prior to injection (GC–MS/MS) and analysis. The mean inter-assay coefficients of variation were 8% for E2, 12% for αPg, and 13% for βPg. The mean intra-assay coefficients of variation were 7% for E2, 12% for αPg and 12% for βPg. Sensitivities of the assays were as follows: E2 and αPg, 0.2 ng/mL; βPg, 10 ng/ mL.

Luteinizing hormone was measured using the commercially available WondFo (Wondfo Biotech Co., Guangzhou, China) Luteinizing Hormone Urinary Test^102^, a validated at-home urine assay. Briefly, the strip was submerged by participants for 5 s in a fresh urine sample and laid horizontally for 5 min before reading. When samples were collected for E2, αPg and βPg, those same samples were used for LH testing. Each strip contains a positive control and a “test” line, indicating if LH is present in the urine at, or over a concentration of 25 MIU/mL^102^. Test results were depicted as either + or – (no quantitative information provided) and were recorded in a personal spreadsheet by the participant. A photograph of each test was taken by participants to ensure accurate reading of the results.

### Inclusion and exclusion criteria for collected cycles

Cycles were included in the premenopausal data set as likely ovulatory by four criteria a) one or more localized days of supra-threshold LH concentration, b) the presence of a rise in E2 (if collected) within typical range prior to or coincident with supra threshold LH, c) a subsequent rise in αPg and βPg (if collected), and d) positive values of DBT deviation, as previously described^98^, within 2 days of surge onset until the end of the cycle (See Supplemental Fig. 1). Cycles without E2, αPg, and βPg data were included by meeting criteria a and d only. Cycles with missing data within sixteen days of the of the LH surge (defined as no HR/HRV/DBT data for a given night) were omitted in order to avoid erroneous estimation of rhythmic power (see *Data Analysis* below). Cycles were defined as “perimenopausal” by the presence of positive LH measured at least every other day across the cycle and age > 45 years. Four such cycles were paired with daily urinary hormone analysis for E2, αPg, and βPg, as described above.

### Data analysis

All code and data used in this paper are available at Open Science Framework (https://osf.io/wzf47/). Code was written in Matlab 2019b, Matlab 2020a and Python 3. Wavelet Transform (WT) code was modified from the Jlab toolbox and from Dr. Tanya Leise^103^. Briefly, data were imported from the Open Humans framework to Python 3, where HR, HRV (RMSSD), and DBT data were extracted. Data were cleaned in Matlab, with any data points outside + /- 4 standard deviations set to the median value of the prior hour, and any points showing near instantaneous change, as defined by a local abs(derivative) > 10^5^ as an arbitrary cutoff, also set to the median value of the previous hour.

Wavelet transformation (WT) was used to assess the structure of ultradian rhythms of DBT, HR, HRV (RMSSD), and circadian rhythms in DBT. As DBT shows high plateaus during the sleeping period, and URs during the day, DBT analyses here were used on data collected during the waking hours (see Supplemental Fig. 2). Conversely, as indicated previously, because the Oura Ring only collects HR and HRV (RMSSD) during sleep, wavelet analyses were restricted to the sleeping window for these metrics. In either case, the excerpted data were compiled from all days of the cycle resulting in one continuous signal representing all days (DBT) or all nights (HR, HRV (RMSSD)). In contrast to Fourier transforms that transform a signal into frequency space without temporal position (i.e., using sine wave components with infinite length), wavelets are constructed with amplitude diminishing to 0 in both directions from center. This property permits frequency strength calculation at a given position. Wavelets can assume many functions (e.g., Mexican hat, square wave, Morse); the present analyses use a Morse wavelet with a low number of oscillations (defined by *β* and *γ*), analogous to wavelets used in previous circadian applications^103^. Morse Wavelet parameters of *β* = 5 and *γ* = 3 describe the frequencies of the two waves superimposed to create the wavelet; Additional values of *β* (3–8) and *γ* (2–5) did not alter the findings (data not shown)^104^. This low number of oscillations enhances detection of contrast and transitions. The band of the wavelet matrix corresponding to 2–5 h rhythms were averaged in order to create a linear representation of UR WT power over time. This band corresponded to the timescale of ultradian rhythmicity observed across physiological systems^14,25,56^. Potential changes to circadian power of DBT (mean power per minute within the 23–25 h band) were additionally assessed prior to extracting days for ultradian-only analyses, but no significant changes across the cycle were detected (see Supplemental Fig. 3). Because WTs exhibit artifacts at the edges of the data being transformed, only the WT of the second through the second to last days of data were analyzed further. To enable comparisons across cycles of different durations, premenopausal cycles were displayed from LH surge onset minus 7 days to LH onset plus 7 days. As perimenopausal individuals had tonically high LH, and therefore no surge onset to which all individuals could be aligned, each cycle’s midpoint was chosen for alignment.

### LH surge anticipation features

Wavelet power in the 2–5 h band was calculated as described above. Extracted bands were smoothed using a daily moving average using the Matlab function “movmean”. The Matlab function “findpeaks” was used to identify peaks as points at which either adjacent point had a lower UR power. This function was run on the negative of the signal to identify troughs. Points at which the derivative of the signal crossed zero, indicating a change in direction of UR power (i.e., either increasing to decreasing or vice versa), were found using the matlab function “diff”. The first time the derivative crossed zero in the cycle (i.e., the first inflection point), excluding the first five days of the cycle, during which LH is very unlikely to rise, was marked as the presence of the first feature for either HRV (RMSSD), DBT, or HR. Following this inflection point, the next peak identified by “findpeaks” was marked as the second feature. These methods of identifying peaks, troughs, and direction changes were used to ensure the diff function was identifying all visually identified peaks.

### Statistical analyses

Descriptive values are reported as means ± daily standard deviations (SD) unless otherwise stated. For statistical comparisons of average ultradian power in premenopausal and perimenopausal cycles, Kruskal Wallis (KW) tests were used instead of ANOVAS to avoid assumptions of normality for any distribution to assess the trend in average UR power leading up to the surge as compared to after the surge. For KW tests, *χ*^2^ and *p* values are listed in the text. One-way repeated measures analysis of variance (rmANOVA) tests were used to compare peak average E2 to other days surrounding the surge, and baseline αPg and βPg (7 days prior to the surge) to other days surrounding the surge. For rmANOVAs, *p* values are listed in the text. Because the dominant trend was an inflection point in UR power followed by a peak, slopes of individual signals were compared rather than raw values at each timepoint. The same tests were applied to individuals, in addition to tests for significance of raw power differences on peak and trough days, using 25 min centered on peaks and troughs, respectively. To avoid multiple comparisons and chance of a type I error, differences between individually-determined peak and trough values of UR WT power found using “findpeaks” were assessed using a KW test, such that each cycle contributed only 1 peak value and 1 trough value (N = 45 data points per group). Figures were formatted in Microsoft PowerPoint 2019 (Microsoft Inc., Redmond, WA) and Adobe Photoshop CS8 (Adobe Inc, San Jose, CA).

## Supplementary information

Supplementary Information.

Supplementary Figures.

## Data Availability

Data and code used to generate the findings in this manuscript can be found on our Open Science Framework page at: https://osf.io/wzf47/

## References

[1] Grimes DA, Gallo MF, Grigorieva V, Nanda K, Schulz KF (2005). Fertility awareness-based methods for contraception: systematic review of randomized controlled trials. Contraception.

[2] Shilaih M (2017). Modern fertility awareness methods: Wrist wearables capture the changes of temperature associated with the menstrual cycle. Biosci. Rep..

[3] Mørch LS (2017). Contemporary hormonal contraception and the risk of breast cancer. N. Engl. J. Med..

[4] Marsden J (2017). Hormonal contraception and breast cancer, what more do we need toknow?. Post Reprod. Health.

[5] Gnoth C, Frank-Herrmann P, Schmoll A, Godehardt E, Freundl G (2002). Cycle characteristics after discontinuation of oral contraceptives. Gynecol. Endocrinol..

[6] Delbarge W (2002). Return to fertility in nulliparous and parous women after removal of the GyneFix intrauterine contraceptive system. *Eur. J. Contracept. Reprod. Health Care Off*. J. Eur. Soc. Contracept..

[7] Bradshaw HK, Mengelkoch S, Hill SE (2020). Hormonal contraceptive use predicts decreased perseverance and therefore performance on some simple and challenging cognitive tasks. Horm. Behav..

[8] Pletzer BA, Kerschbaum HH (2014). 50 years of hormonal contraception—time to find out, what it does to our brain. Front. Neurosci..

[9] Skovlund CW, Mørch LS, Kessing LV, Lange T, Lidegaard Ø (2018). Association of hormonal contraception with suicide attempts and suicides. Am. J. Psychiatry.

[10] Skovlund CW, Mørch LS, Kessing LV, Lidegaard Ø (2016). Association of hormonal contraception with depression. JAMA Psychiatry.

[11] Brar TK, Singh KD, Kumar A (2015). Effect of different phases of menstrual cycle on heart rate variability (HRV). J. Clin. Diagn. Res. JCDR.

[12] Smarr BL, Zucker I, Kriegsfeld LJ (2016). Detection of successful and unsuccessful pregnancies in mice within hours of pairing through frequency analysis of high temporal resolution core body temperature data. PLoS ONE.

[13] Smarr BL, Grant AD, Zucker I, Prendergast BJ, Kriegsfeld LJ (2017). Sex differences in variability across timescales in BALB/c mice. Biol. Sex Differ..

[14] Grant AD, Wilsterman K, Smarr BL, Kriegsfeld LJ (2018). Evidence for a coupled oscillator model of endocrine ultradian rhythms. J. Biol. Rhythms.

[15] Baerwald AR, Adams GP, Pierson RA (2012). Ovarian antral folliculogenesis during the human menstrual cycle: a review. Hum. Reprod. Update.

[16] Ferreira SR, Motta AB (2018). Uterine function: from normal to polycystic ovarian syndrome alterations. Curr. Med. Chem..

[17] Simonneaux V, Bahougne T, Angelopoulou E (2017). Daily rhythms count for femalefertility. Best Pract. Res. Clin. Endocrinol. Metab..

[18] Robker RL, Hennebold JD, Russell DL (2018). Coordination of ovulation and oocyte maturation: a good egg at the right time. Endocrinology.

[19] Zhang S (2020). Changes in sleeping energy metabolism and thermoregulationduringmenstrual cycle. Physiol. Rep..

[20] Yeung EH (2010). Longitudinal study of insulin resistance and sex hormones over the menstrual cycle: the biocycle study. J. Clin. Endocrinol. Metab..

[21] Tada Y (2017). The impact of menstrual cycle phases on cardiac autonomic nervous system activity: an observational study considering lifestyle (diet, physical activity, and sleep) among female college students. J. Nutr. Sci. Vitaminol. (Tokyo).

[22] Stanford JB (2015). Revisiting the fertile window. Fertil. Steril..

[23] Shannahoff-Khalsa DS, Kennedy B, Yates FE, Ziegler MG (1996). Ultradian rhythms of autonomic, cardiovascular, and neuroendocrine systems are related in humans. Am. J. Physiol..

[24] Shannahoff-Khalsa DS, Kennedy B, Yates FE, Ziegler MG (1997). Low-frequency ultradian insulin rhythms are coupled to cardiovascular, autonomic, and neuroendocrine rhythms. Am. J. Physiol. Regul. Integr. Comp. Physiol..

[25] Brandenberger G, Simon C, Follenius M (1987). Ultradian endocrine rhythms: a multioscillatory system. J. Interdiscip. Cycle Res..

[26] Zavala E (2019). Mathematical modelling of endocrine systems. Trends Endocrinol. Metab..

[27] Clarke IJ, Thomas GB, Yao B, Cummins JT (1987). GnRH secretion throughout the ovine estrous cycle. Neuroendocrinology.

[28] Gore AC, Windsor-Engnell BM, Terasawa E (2004). Menopausal increases in pulsatile gonadotropin-releasing hormone release in a nonhuman primate (*Macaca mulatta*). Endocrinology.

[29] Moenter SM, Caraty A, Locatelli A, Karsch FJ (1991). Pattern of gonadotropin-releasing hormone (GnRH) secretion leading up to ovulation in the ewe: existence of a preovulatory GnRH surge. Endocrinology.

[30] Backstrom C, McNeilly AS, Leask R, Baird D (1982). Pulsatile secretion of LH, FSH, Prolactin, oestradiol, and progesterone during the human menstrual cycle. Clin. Endocrinol. (Oxf.).

[31] Rossmanith WG (1990). Pulsatile cosecretion of estradiol and progesterone by the midluteal phase corpus luteum: temporal link to luteinizing hormone pulses. J. Clin. Endocrinol. Metab..

[32] Vugt DAV, Lam NY, Ferin M (1984). Reduced frequency of pulsatile luteinizing hormone secretion in the luteal phase of the rhesus monkey involvement of endogenous opiates. Endocrinology.

[33] Booth RA (1996). Mode of pulsatile follicle-stimulating hormone secretion in gonadal hormone-sufficient and-deficient women–a clinical research center study. J. Clin. Endocrinol. Metab..

[34] Genazzani AD (1993). FSH secretory pattern and degree of concordance with LH in amenorrheic, fertile, and postmenopausal women. Am. J. Physiol. - Endocrinol. Metab..

[35] Pincus SM, Padmanabhan V, Lemon W, Randolph J, Rees Midgley A (1998). Follicle- stimulating hormone is secreted more irregularly than luteinizing hormone in both humans and sheep. J. Clin. Invest..

[36] Yen SSC, Tsai CC, Naftolin F, Vandenberg G, Ajabor L (1972). Pulsatile patterns of gonadotropin release in subjects with and without ovarian function. J. Clin. Endocrinol. Metab..

[37] Licinio J (1998). Synchronicity of frequently sampled, 24-h concentrations of circulating leptin, luteinizing hormone, and estradiol in healthy women. Proc. Natl. Acad. Sci. USA.

[38] Soules MR, Clifton DK, Cohen NL, Bremner WJ, Steiner RA (1989). Luteal phase deficiency: abnormal gonadotropin and progesterone secretion patterns. J. Clin. Endocrinol. Metab..

[39] Veldhuis JD (1988). Physiological profiles of episodic progesterone release during the midluteal phase of the human menstrual cycle. J. Clin. Endocrinol. Metab..

[40] Filicori M, Butler JP, Crowley WF (1984). Neuroendocrine regulation of the corpus luteum in the human. Evidence for pulsatile progesterone secretion. J. Clin. Invest..

[41] Genazzani AD, Guardabasso V, Petraglia F, Genazzani AR (1991). Specific concordance index defines the physiological lag between LH and progesterone in women during the midluteal phase of the menstrual cycle. Gynecol. Endocrinol..

[42] Nóbrega LHC (2009). Analysis of testosterone pulsatility in women with ovulatory menstrual cycles. Arq. Bras. Endocrinol. Metabol..

[43] Healy DL, Schenken RS, Lynch A, Williams RF, Hodgen GD (1984). Pulsatile progesterone secretion: its relevance to clinical evaluation of corpus luteum function. Fertil. Steril..

[44] Shechter A, Boudreau P, Varin F, Boivin DB (2011). Predominance of distal skin temperature changes at sleep onset across menstrual and circadian phases. J. Biol. Rhythms.

[45] Leicht AS, Hirning DA, Allen GD (2003). Heart rate variability and endogenous sex hormones during the menstrual cycle in young women. Exp. Physiol..

[46] Chen, W., Kitazawa, M. & Togawa, T. HMM-based estimation of menstrual cycle from skin temperature during sleep. In *2008 30th Annual International Conference of the IEEE Engineering in Medicine and Biology Society* **2008**, 1635–1638 (2008). 10.1109/IEMBS.2008.4649487. 10.1109/IEMBS.2008.4649487 19162990

[47] Charkoudian N, Hart ECJ, Barnes JN, Joyner MJ (2017). Autonomic control ofbody temperature and blood pressure: influences of female sex hormones. Clin. Auton. Res..

[48] de Zambotti M, Willoughby AR, Sassoon SA, Colrain IM, Baker FC (2015). Menstrual cycle-related variation in physiological sleep in women in the early menopausal transition. J. Clin. Endocrinol. Metab..

[49] de Zambotti M, Nicholas CL, Colrain IM, Trinder JA, Baker FC (2013). Autonomic regulation across phases of the menstrual cycle and sleep stages in women with premenstrual syndrome and healthy controls. Psychoneuroendocrinology.

[50] Stein PK (2006). Circadian and ultradian rhythms in heart rate variability. Biomed. Tech. (Berl).

[51] Stein PK (2006). Circadian and ultradian rhythms in cardiac autonomic modulation. Conf. Proc. Annu. Int. Conf. IEEE Eng. Med. Biol. Soc. IEEE Eng. Med. Biol. Soc. Annu. Conf..

[52] Visrutha K, Harini N, Ganaraja B, Pavanchand A, Veliath S (2012). A study of cardiac autonomic control and pulmonary functions in different phases of menstrual cycle. Int. J. Appl. Biol. Pharm. Technol..

[53] Buxton CL, Atkinson WB (1948). Hormonal factors involved in the regulation of basal body temperature during the menstrual cycle and pregnancy. J. Clin. Endocrinol. Metab..

[54] Fredholm BB, Johansson S, Wang Y-Q (2011). Adenosine and the regulation of metabolism and body temperature. Adv. Pharmacol. San Diego Calif.

[55] Stuckey MI, Kiviniemi A, Gill DP, Shoemaker JK, Petrella RJ (2015). Associations between heart rate variability, metabolic syndrome risk factors, and insulin resistance. Appl. Physiol. Nutr. Metab. Physiol. Appl. Nutr. Metab..

[56] Goh GH, Maloney SK, Mark PJ, Blache D (2019). Episodic ultradian events—ultradian rhythms. Biology.

[57] Sato N, Miyake S, Akatsu J, Kumashiro M (1995). Power spectral analysis of heart rate variability in healthy young women during the normal menstrual cycle. Psychosom. Med..

[58] Uckuyu A, Toprak E, Ciftci O, Ciftci FC (2013). The fluctuation in the heart rate variability throughout ovulation induction cycle: is the case different in polycystic ovary syndrome?. Gynecol. Obstet..

[59] Yildirir A, Aybar F, Kabakci G, Yarali H, Oto A (2006). Heart rate variability in young women with polycystic ovary syndrome. Ann. Noninvasive Electrocardiol..

[60] Kräuchi K (2014). Diurnal and menstrual cycles in body temperature are regulated differently: a 28-day ambulatory study in healthy women with thermal discomfort of cold extremities and controls. Chronobiol. Int..

[61] Uchida Y, Atsumi K, Takamata A, Morimoto K (2019). The effect of menstrual cycle phase on foot skin temperature during mild local cooling in young women. J. Physiol. Sci. JPS.

[62] Baker FC, Driver HS (2007). Circadian rhythms, sleep, and the menstrual cycle. Sleep Med..

[63] Bauman JE (1981). Basal body temperature: unreliable method of ovulation detection. Fertil. Steril..

[64] Quagliarello J, Arny M (1986). Inaccuracy of basal body temperature charts in predicting urinary luteinizing hormone surges. Fertil. Steril..

[65] de Zambotti M, Rosas L, Colrain IM, Baker FC (2017). The sleep of the ring: comparison of the ŌURA sleep tracker against polysomnography. Behav. Sleep. Med..

[66] Hasselberg MJ, McMahon J, Parker K (2013). The validity, reliability, and utility of the iButton for measurement of body temperature circadian rhythms in sleep/wake research. Sleep Med..

[67] Gear, A. J. C. H. J. C. H. Wearables are totally failing the people who need them most. *WIRED* https://www.wired.com/2014/11/where-fitness-trackers-fail/.

[68] Epstein DA (2017). Examining menstrual tracking to inform the design of personal informatics tools. Proc. SIGCHI Conf. Hum. Factors Comput. Syst. CHI Conf..

[69] Bashan A, Bartsch RP, Kantelhardt JW, Havlin S, Ivanov PC (2012). Network physiology reveals relations between network topology and physiological function. Nat. Commun..

[70] Bartsch RP, Liu KKL, Bashan A, Ivanov PC (2015). Network physiology: how organ systems dynamically interact. PLoS ONE.

[71] Direito A, Bailly S, Mariani A, Ecochard R (2013). Relationships between the luteinizing hormone surge and other characteristics of the menstrual cycle in normally ovulating women. Fertil. Steril..

[72] Williams H, Dacks PA, Rance NE (2010). An improved method for recording tail skin temperature in the rat reveals changes during the Estrous cycle and effects of ovarian steroids. Endocrinology.

[73] Coyne MD, Kesick CM, Doherty TJ, Kolka MA, Stephenson LA (2000). Circadian rhythm changes in core temperature over the menstrual cycle: method for noninvasive monitoring. Am. J. Physiol.-Regul. Integr. Comp. Physiol..

[74] Solomon SJ, Kurzer MS, Calloway DH (1982). Menstrual cycle and basal metabolic rate in women. Am. J. Clin. Nutr..

[75] Sanchez-Alavez M, Alboni S, Conti B (2011). Sex- and age-specific differences in core body temperature of C57Bl/6 mice. Age Dordr. Neth..

[76] Shechter A, Boivin DB (2010). Sleep, hormones, and circadian rhythms throughout the menstrual cycle in healthy women and women with premenstrual dysphoric disorder. Int. J. Endocrinol..

[77] Szymusiak R (2018). Body temperature and sleep. Handb. Clin. Neurol..

[78] Henane R, Buguet A, Roussel B, Bittel J (1977). Variations in evaporation and body temperatures during sleep in man. J. Appl. Physiol..

[79] Schmalenberger KM (2019). A systematic review and meta-analysis of within-person changes in cardiac vagal activity across the menstrual cycle: implications for female health and future studies. J. Clin. Med..

[80] Jain A (2007). Pulsatile luteinizing hormone amplitude and progesterone metabolite excretion are reduced in obese women. J. Clin. Endocrinol. Metab..

[81] van Leckwyck M (2016). Decreasing insulin sensitivity in women induces alterations in LH pulsatility. J. Clin. Endocrinol. Metab..

[82] Lessey BA, Young SL (2019). What exactly is endometrial receptivity?. Fertil. Steril..

[83] Faust L (2019). Findings from a mobile application-based cohort are consistent with established knowledge of the menstrual cycle, fertile window, and conception. Fertil. Steril..

[84] Setton R, Tierney C, Tsai T (2016). The accuracy of web sites and cellular phone applications in predicting the fertile window. Obstet. Gynecol..

[85] Su H-W, Yi Y-C, Wei T-Y, Chang T-C, Cheng C-M (2017). Detection of ovulation, a review of currently available methods. Bioeng. Transl. Med..

[86] Keulers MJ, Hamilton CJCM, Franx A, Evers JLH, Bots RSGM (2007). The length of the fertile window is associated with the chance of spontaneously conceiving an ongoing pregnancy in subfertile couples. Hum. Reprod. Oxf. Engl..

[87] Ecochard R, Duterque O, Leiva R, Bouchard T, Vigil P (2015). Self-identification of the clinical fertile window and the ovulation period. Fertil. Steril..

[88] Wilcox AJ, Dunson D, Baird DD (2000). The timing of the “fertile window” in the menstrual cycle: day specific estimates from a prospective study. BMJ.

[89] Moghissi KS (1976). Accuracy of basal body temperature for ovulation detection. Fertil. Steril..

[90] Newman M, Pratt SM, Curran DA, Stanczyk FZ (2019). Evaluating urinary estrogen and progesterone metabolites using dried filter paper samples and gas chromatography with tandem mass spectrometry (GC–MS/MS). BMC Chem..

[91] Freis A (2018). Plausibility of menstrual cycle apps claiming to support conception. Front. Public Health.

[92] Renaud RL (1980). Echographic study of follicular maturation and ovulation during the normal menstrual cycle. Fertil. Steril..

[93] Queenan JT (1980). Ultrasound scanning of ovaries to detect ovulation in women. Fertil. Steril..

[94] Polan ML (1982). Abnormal ovarian cycles as diagnosed by ultrasound and serum estradiol levels. Fertil. Steril..

[95] Choe, E. K., Lee, N. B., Lee, B., Pratt, W. & Kientz, J. A. Understanding quantified-selfers’ practices in collecting and exploring personal data. In *Proceedings of the 32nd annual ACM conference on human factors in computing systems*, 1143–1152 (2014).

[96] Grant AD, Wolf GI, Nebeker C (2019). Approaches to governance of participant-led research: a qualitative case study. BMJ Open.

[97] Vayena E, Tasioulas J (2013). Adapting standards: Ethical oversight of participant-led health research. PLoS Med..

[98] Maijala A, Kinnunen H, Koskimäki H, Jämsä T, Kangas M (2019). Nocturnal finger skin temperature in menstrual cycle tracking: ambulatory pilot study using a wearable Oura ring. BMC Womens Health.

[99] Grant AD, Wolf GI (2019). Free-living humans cross cardiovascular disease risk categories due to daily rhythms in cholesterol and triglycerides. J. Circadian Rhythms.

[100] Greshake Tzovaras B (2019). Open humans: a platform for participant-centered research and personal data exploration. GigaScience.

[101] Roos J (2015). Monitoring the menstrual cycle: Comparison of urinary and serum reproductive hormones referenced to true ovulation. Eur. J. Contracept. Reprod. Health Care Off. J. Eur. Soc. Contracept..

[102] Barron ML, Vanderkolk K, Raviele K (2018). Finding the fertile phase: low-cost luteinizing hormone sticks versus electronic fertility monitor. MCN. Am. J. Matern. Child Nurs..

[103] Leise TL (2013). Wavelet analysis of circadian and ultradian behavioral rhythms. J. Circadian Rhythms.

[104] Lilly JM, Olhede SC (2012). Generalized morse wavelets as a superfamily of analytic wavelets. IEEE Trans. Signal Process..

