# Supplementary material for: Ultradian rhythms in heart rate variability and distal body temperature anticipate onset of the luteinizing hormone surge
Source: Sci Rep. 2020 Nov 23;10:20378. doi: 10.1038/s41598-020-76236-6 (PMC7683606; doi:10.1038/s41598-020-76236-6)
Supplement: Supplementary file 1 — Supplementary Information. [file 41598_2020_76236_MOESM1_ESM.docx]

Revised manuscript sent to *Scientific Reports* on 10/11/2020

**Ultradian Rhythms in Heart Rate Variability and Distal Body Temperature**

**Anticipate the Luteinizing Hormone Surge Onset**

**Azure D. Grant^1^, Mark Newman^2^, Lance J. Kriegsfeld^1,3,4,5^**

^1^The Helen Wills Neuroscience Institute, University of California, Berkeley, CA, 94720 ^2^Precision Analytical, McMinnville, OR, 97128; ^3^Department of Psychology, University of California, Berkeley, CA 94720; ^4^Department of Integrative Biology, University of California, Berkeley, CA 94720; ^5^Graduate Group in Endocrinology, University of California, Berkeley, CA 94720

**Brief Title:** Ultradian Rhythms in HRV and temperature anticipate the LH Surge

**Key Words:** LH Surge, HRV, Fertility Awareness, Signal Processing, Menstrual Cycle, Skin Temperature, Wearable Device

**Address Correspondence to:**

Lance J. Kriegsfeld, PhD

Department of Psychology, Integrative Biology,

Graduate Group in Endocrinology and

The Helen Wills Neuroscience Institute

2121 Berkeley Way

University of California, Berkeley CA 94720

**E-mail**[: kriegsfeld@berkeley.edu](mailto:kriegsfeld@berkeley.edu)

**Phone**: 510-642-5148

**Fax**: 510 642 5293

Supplemental Figure Legends

***Supplemental Figure 1*. Sleep timing, duration, latency, breathing Rate and temperature deviation do not anticipate the LH surge.** Bedtime (A), wake time (B), sleep duration (C) and sleep latency (D) do not show reliable changes relative to LH surge onset in premenopausal individuals. Breathing rate (E) and nightly temperature deviation (F, see methods) do not anticipate the surge, but exhibit an upward trend following the surge, as previously described (Maijala *et al.*, 2019).

***Supplemental Figure 2*. Heart rate ultradian power does not anticipate LH surge onset.** Mean sleeping heart rate ultradian power ± standard deviation for premenopausal cycles (A) within one week of LH surge onset and perimenopausal cycles (C) within one week of mid cycle. HR ultradian fluctuations do not anticipate the LH surge (p>0.05) but exhibit a significant elevation 2-3 days after the surge (χ^2^ =0.3, *p*=0.04 ). Representative individual examples of HR ultradian power within one week of LH surge onset or mid cycle in premenopausal (B) and perimenopausal (D) cycles, respectively.

***Supplemental Figure 3.* Circadian power of body temperature does not change stereotypically around LH surge onset.** Mean DBT circadian power ± standard deviation for premenopausal cycles (A) within one week of LH surge onset and perimenopausal cycles (C) within one week of mid cycle do not exhibit significant stereotyped fluctuations relative to LH surge onset or mid cycle. Individuals varied widely (examples B and D).

***Supplemental Figure 4*. Linear average of DBT relative to LH surge onset in premenopausal and mid cycle in perimenopausal individuals.** Mean (A), ± standard deviation (shaded) of linear DBT around LH surge onset. Perimenopausal mean (B) ± standard deviation (shaded) of linear DBT surrounding mid cycle (note two perimenopausal cycles were very short, with only 6 days after midcycle occurring before next menses), and individual example (C). Individual example of DBT around LH surge onset (D) and zoomed window in this individual from LH-3 days to LH illustrating the presence of high amplitude URs during the day and a relatively high plateau during sleep (E).

***Supplemental Figure 5.* Linear average of sleeping HR relative to LH surge onset in premenopausal and mid cycle in perimenopausal individuals.** Premenopausal average (A), ± standard deviation (shaded) of linear sleeping HR surrounding LH surge onset, and individual example (B). Perimenopausal average (C) ± standard deviation (shaded) of linear HR surrounding mid cycle, and individual example (D).

***Supplemental Figure 6*. Linear average of sleeping HRV (RMSSD) relative to LH Surge onset in premenopausal and mid cycle in perimenopausal individuals.** Premenopausal average (A), ± standard deviation (shaded) of linear sleeping HRV surrounding LH surge onset, and individual example (B). Perimenopausal average (C) ± standard deviation (shaded) of linear HRV surrounding mid cycle, and individual example (D).
