# Supplementary figures and images for: Ultradian rhythms in heart rate variability and distal body temperature anticipate onset of the luteinizing hormone surge
Source: Sci Rep. 2020 Nov 23;10:20378. doi: 10.1038/s41598-020-76236-6 (PMC7683606; doi:10.1038/s41598-020-76236-6)

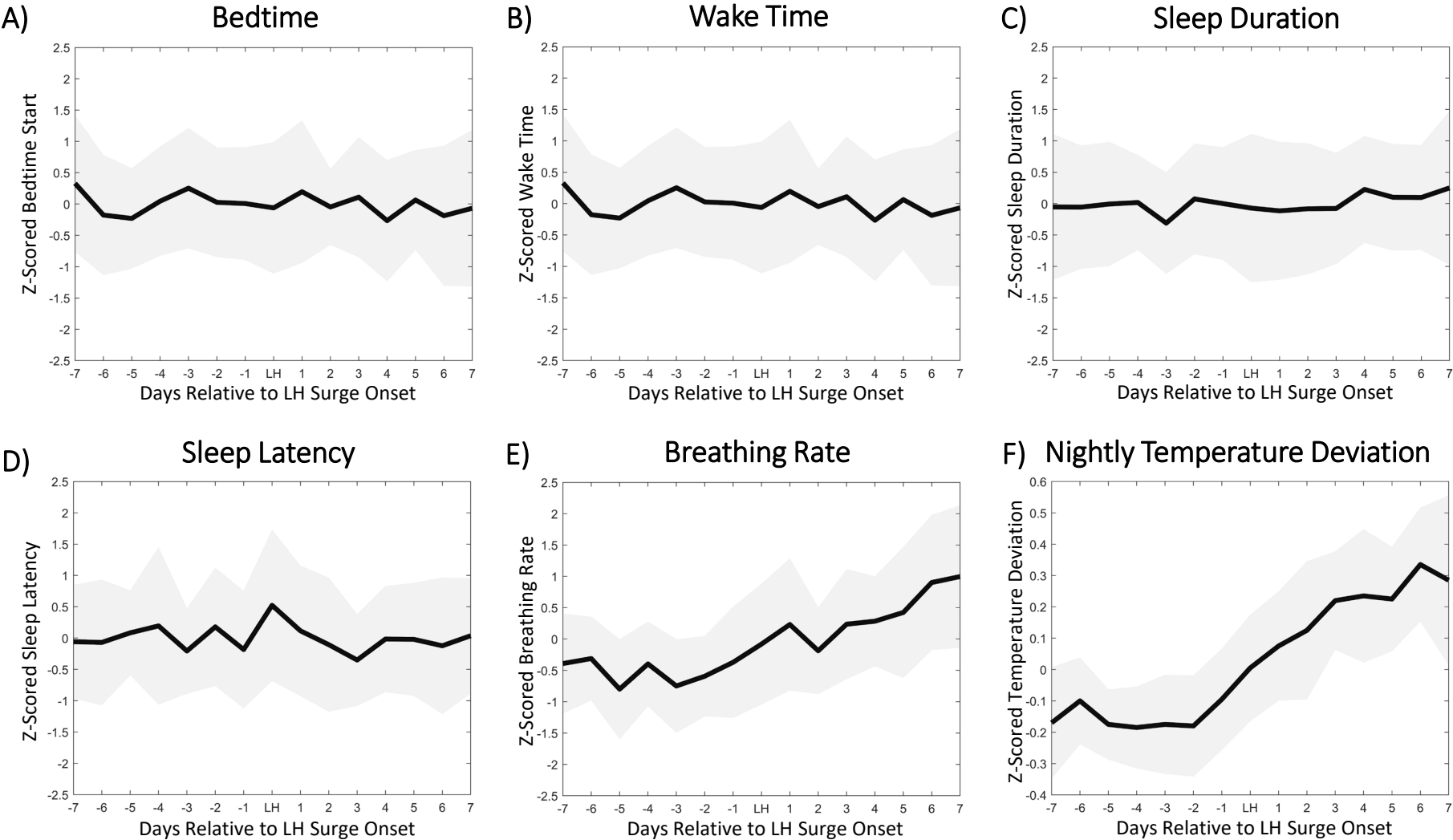

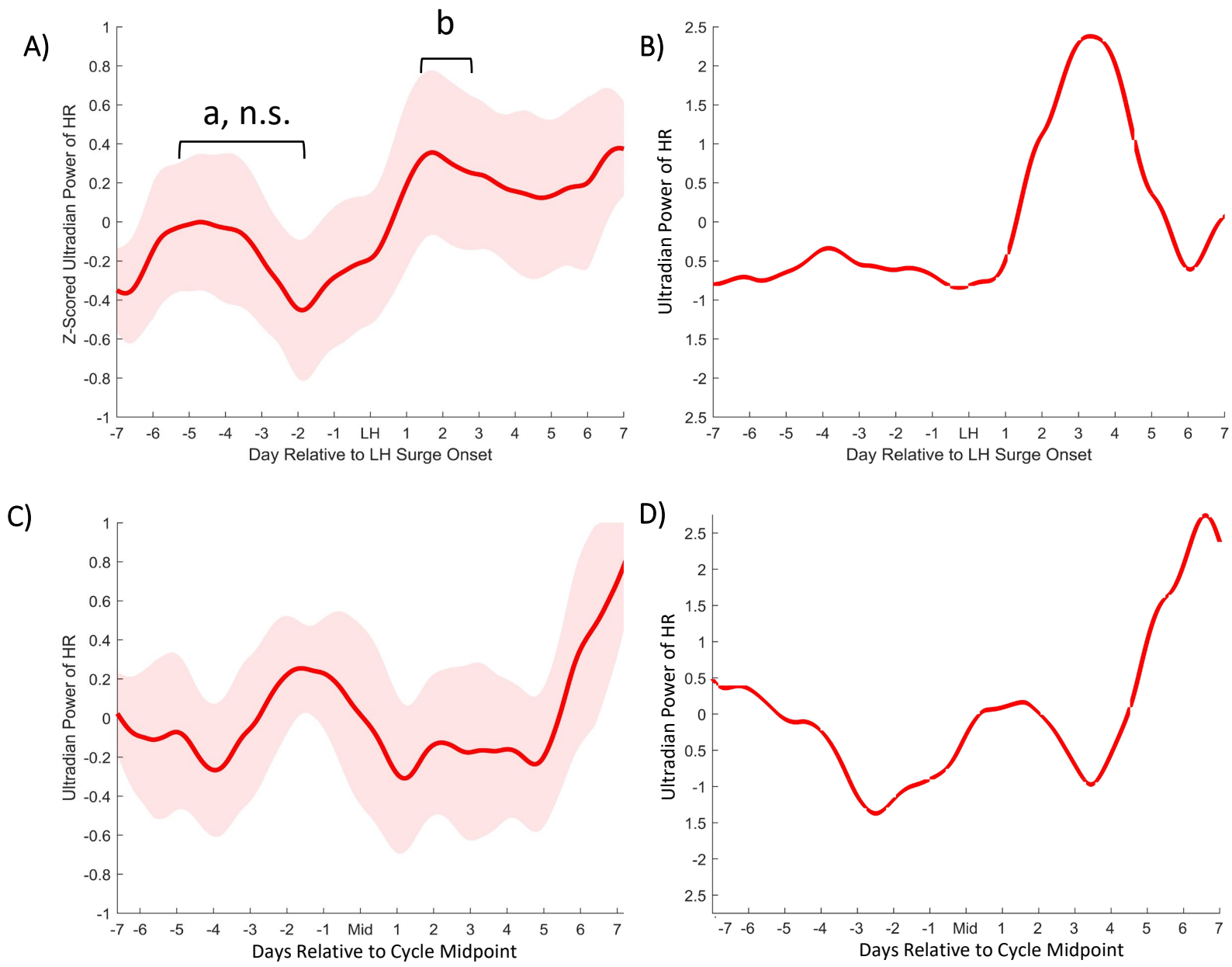

A)

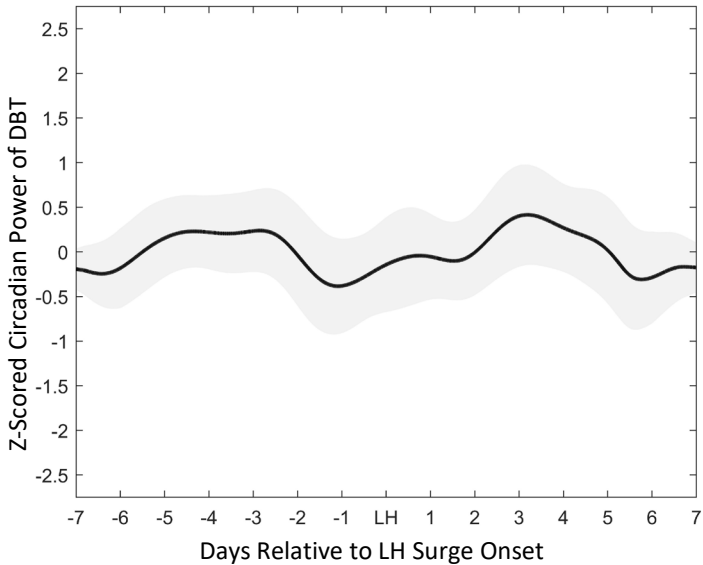

B)

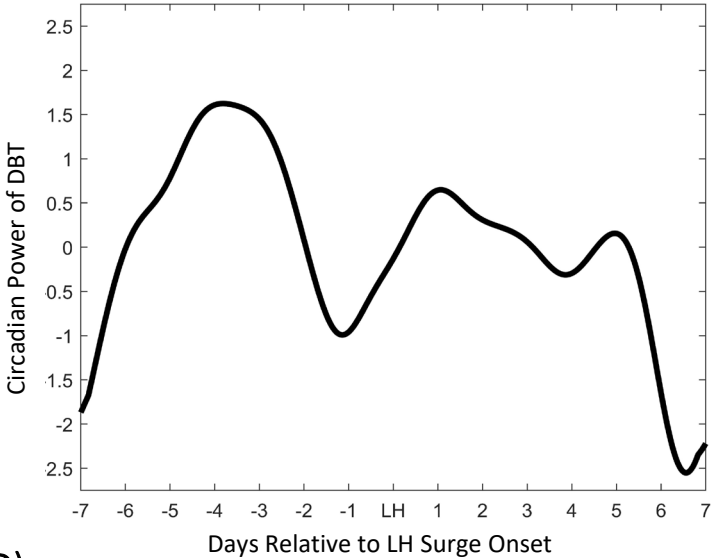

C)

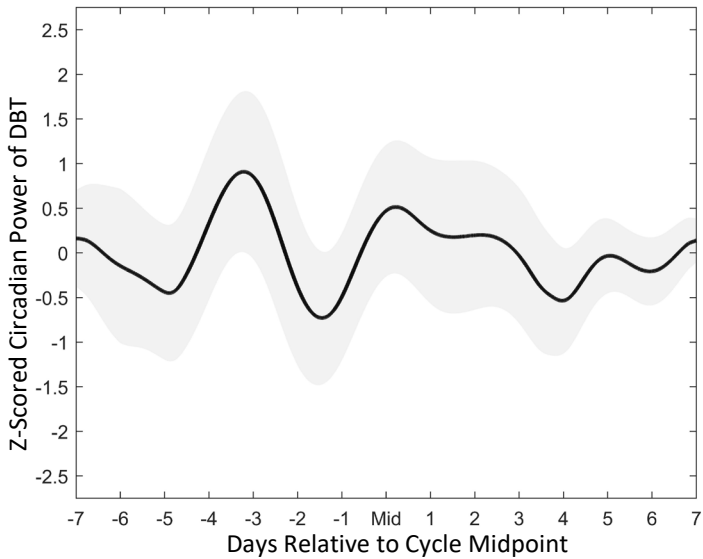

D)

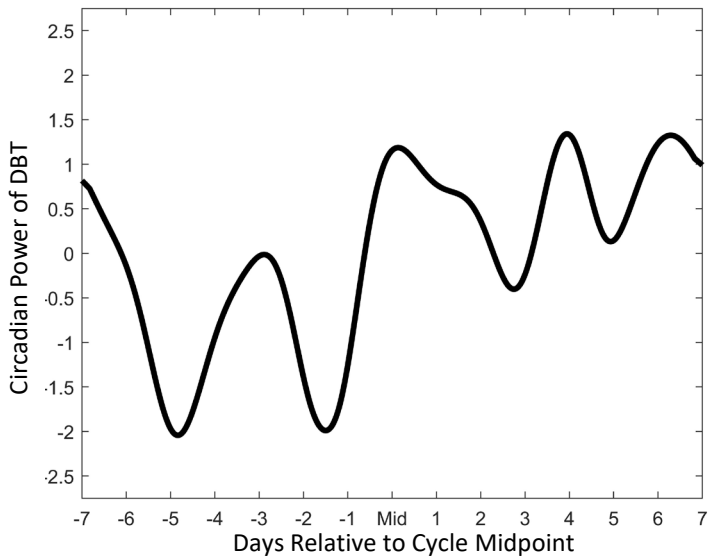

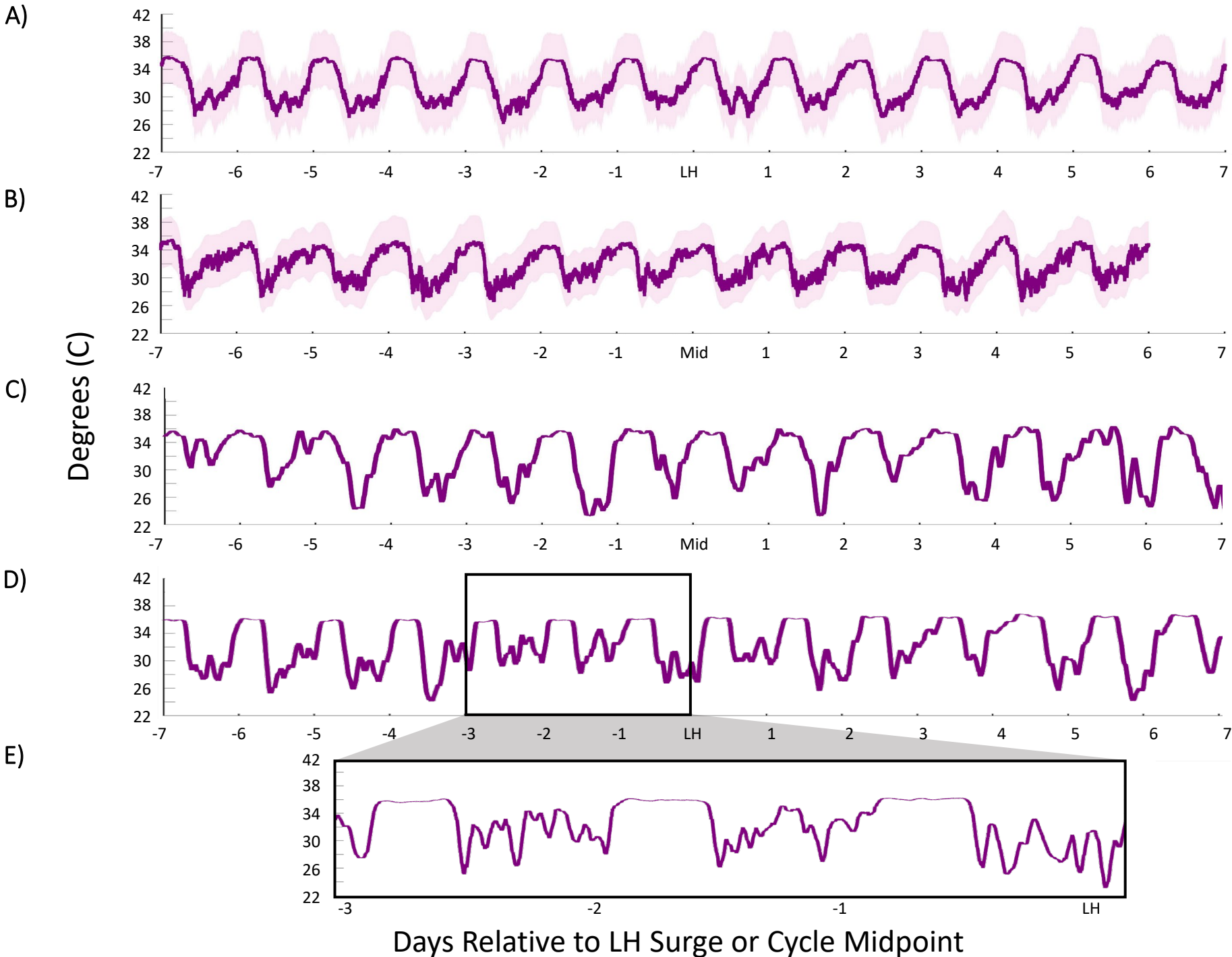

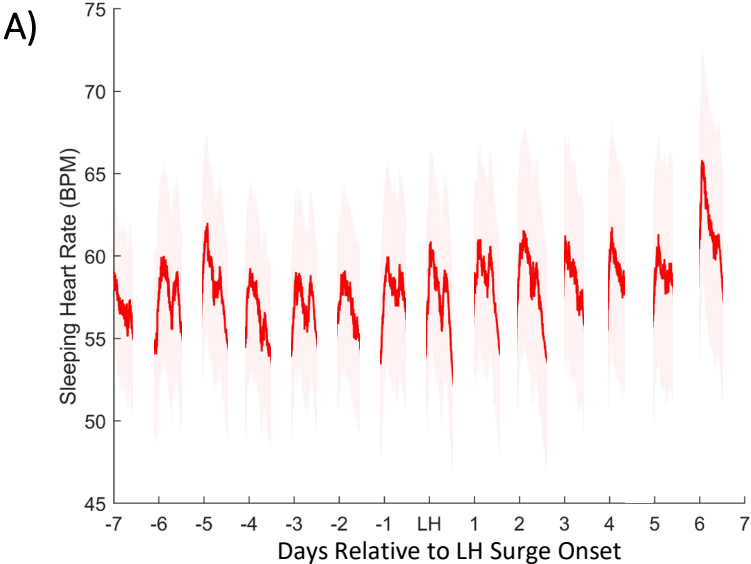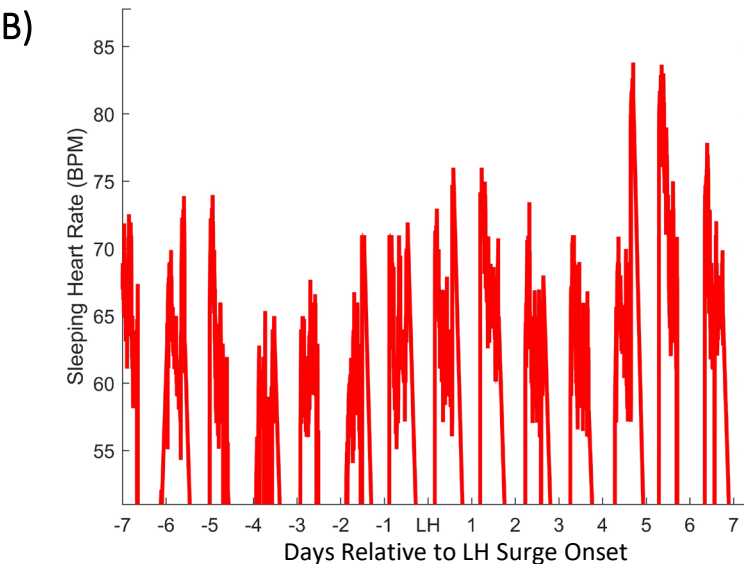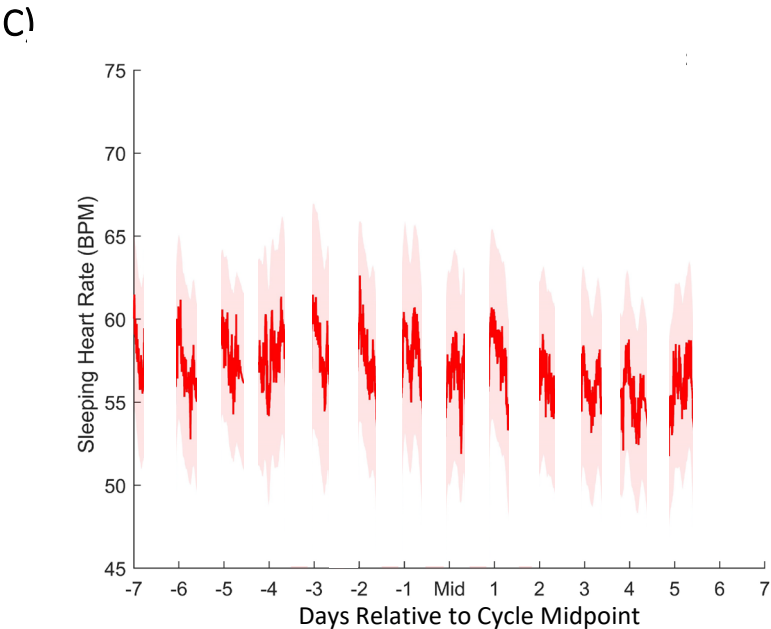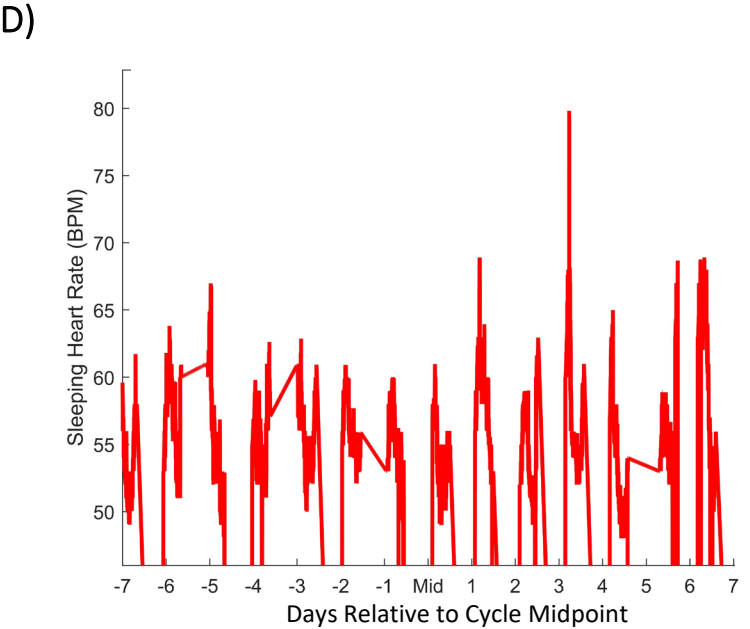

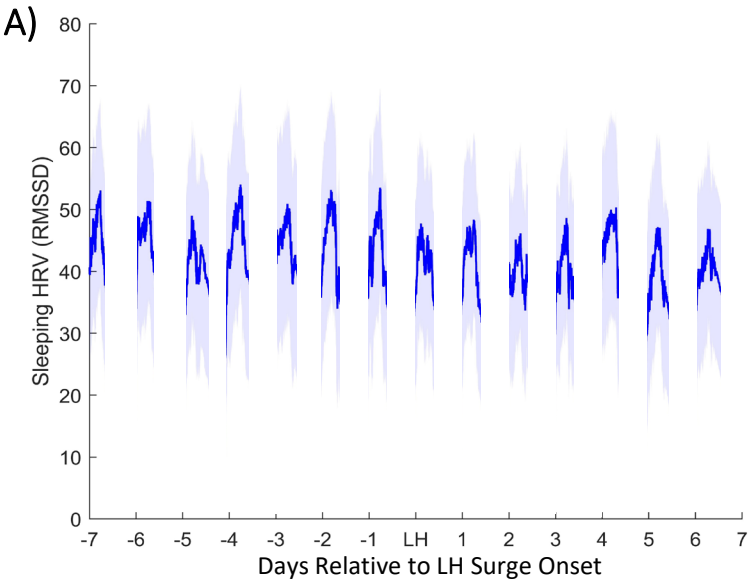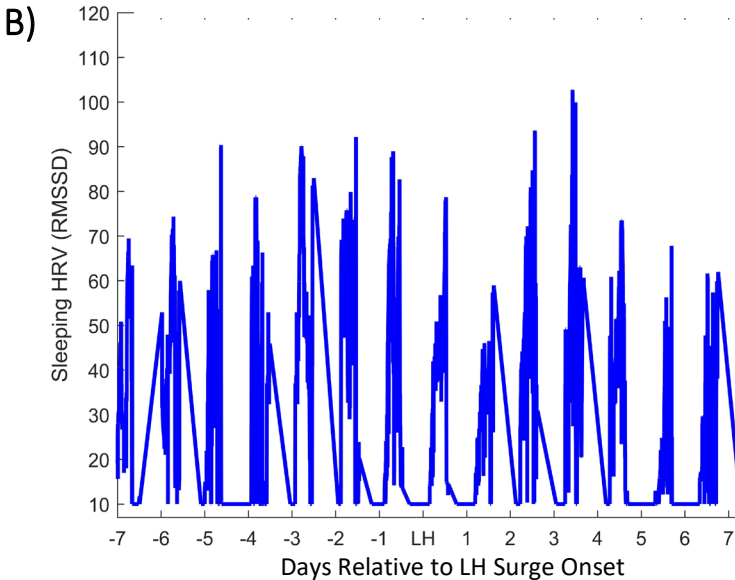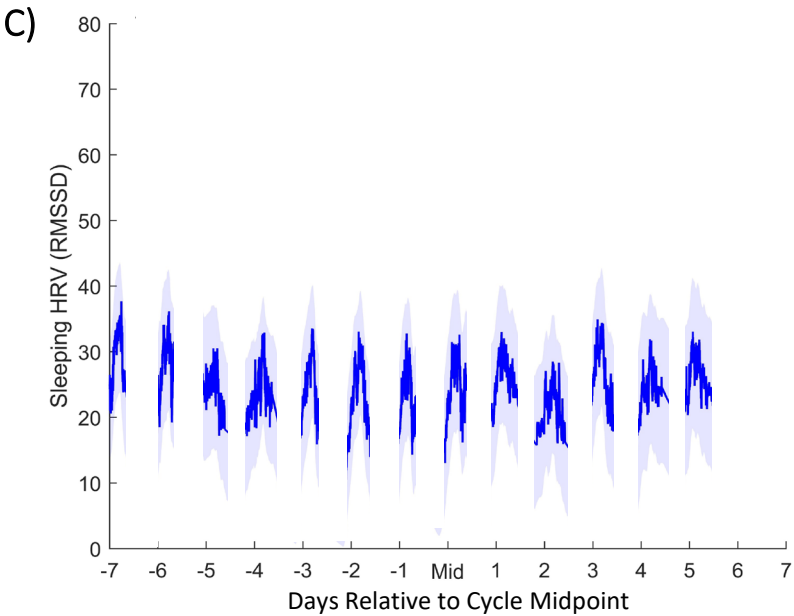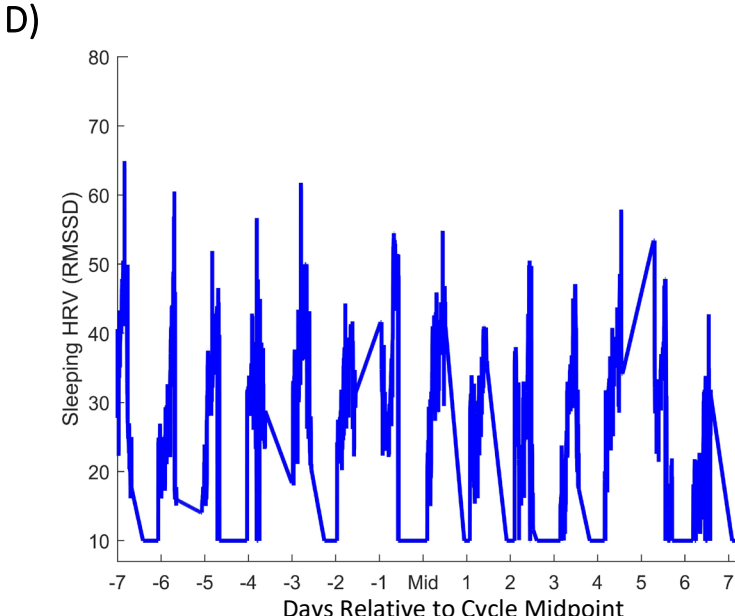

Supplement: Supplementary file 2 — Supplementary Figures. [file 41598_2020_76236_MOESM2_ESM.pdf]
